# Assessment of Hearing Dysfunction in Patients With Graves’ Disease and Thyroid Eye Disease Without or With Teprotumumab

**DOI:** 10.1210/clinem/dgae560

**Published:** 2024-08-14

**Authors:** Terry J Smith, Robert J Holt, Qianhong Fu, Anahita Qashqai, Naina Barretto, Elizabeth Conrad, Jason A Brant

**Affiliations:** Department of Ophthalmology and Visual Sciences, W. K. Kellogg Eye Center, and Department of Internal Medicine, University of Michigan Medical School, Ann Arbor, MI 48105, USA; Medical Affairs, Amgen Inc, Thousand Oaks, CA 91320, USA; Medical Affairs, Amgen Inc, Thousand Oaks, CA 91320, USA; Medical Affairs, Amgen Inc, Thousand Oaks, CA 91320, USA; Medical Affairs, Amgen Inc, Thousand Oaks, CA 91320, USA; Medical Affairs, Amgen Inc, Thousand Oaks, CA 91320, USA; Department of Otorhinolaryngology—Head and Neck Surgery, University of Pennsylvania, Philadelphia, PA 19104, USA

**Keywords:** hearing loss, teprotumumab, thyroid eye disease, Graves’ disease

## Abstract

**Context:**

Thyroid eye disease (TED) negatively affects quality of life. TED occurs predominantly in Graves’ disease (GD). Teprotumumab improves TED but concern for hearing adverse events (AEs) has emerged. Hearing dysfunction is reported in thyroid autoimmune disease but the background prevalence in GD/TED without teprotumumab remains uncertain.

**Objective:**

This work aimed to quantify ear-related diagnostic codes/hearing AEs in GD, TED, and patients receiving teprotumumab by examining medical claims and clinical trials.

**Methods:**

Deidentified claims for ear/labyrinth-related International Classification of Disorders, Tenth Revision codes (KOMODO) were examined in GD patients without TED, and TED patients without/with teprotumumab treatment. Hearing AE incidence/severity was evaluated in teprotumumab clinical trials. Graves’ Ophthalmopathy Quality of Life questionnaire (GO-QOL) scores were compared in teprotumumab TED trial patients without/with hearing AEs.

**Results:**

GD (469 720), TED (38 566) and teprotumumab-treated (967) patients were identified in the claims database. Ear-related codes (including those not specific for hearing) occurred in 24% GD, 33% TED, and 32% teprotumumab-treated patients. “Sensorineural hearing loss bilateral” was most frequent: 7% (32 961/469 720) GD, 11.1% (4279/38 566) TED, and 10.8% (104/967) teprotumumab patients. Before teprotumumab use, 17.1% (165) patients had ear-related codes, while 10.1% (98) had new ear-related codes post treatment.

Eight teprotumumab oncology trials revealed 8.1% (51/633) had ear/labyrinth disorders with 2.1% (13) considered study-drug-related and 3.8% (24) hearing impairment/tinnitus-related AEs with 1.3% (8) considered study-drug-related. Similar rates occurred in TED trials.

GO-QOL improved in teprotumumab-treated patients without/with hearing AEs. Incidence/severity was consistent across patients with chronic and acute TED.

**Conclusion:**

These analyses indicate similar prevalence of hearing claims in patients with GD/TED alone as following teprotumumab treatment. Future analyses of incremental hearing risk from teprotumumab should use a priori study designs accounting for background hearing dysfunction in patients with GD/TED.

Thyroid eye disease (TED, also known as Graves’ orbitopathy or thyroid-associated ophthalmopathy) is an autoimmune disease involving orbital fat/connective tissue expansion and remodeling (1). TED is characterized by progressive strabismus, diplopia, and proptosis (1). Up to 90% of patients with TED also manifest Graves’ disease (GD) (2). TED can cause a severe and persistent negative effect on patient quality of life (QOL) (3, 4).

Insulin-like growth factor-1 receptor (IGF-IR) plays a central role in the pathogenesis of TED, in part by virtue of its interactions with the thyrotropin receptor, the central autoantigen in thyroidal dysfunction associated with GD (5). Molecular studies implicating the involvement of IGF-IR in TED (6) led to the development of teprotumumab as a therapy for the disease, based on 2 pivotal clinical trials (7, 8) and a key extension study (9). In the registrational trials, teprotumumab, an IGF-IR inhibitory antibody, demonstrated efficacy in reducing inflammation, proptosis, and diplopia in patients with acute TED (clinical activity score [CAS] ≥ 4, duration ≤9 months) (10). Since teprotumumab's approval in 2020, it remains the only medical therapy approved by the US Food and Drug Administration (FDA) for the treatment of TED, regardless of disease activity/severity/duration.

IGF-IR exhibits involvement in a vast array of physiological functions, including those surrounding cellular metabolism, immunity, and neurologic senses. Hearing abnormalities were reported by about 10% of patients receiving teprotumumab in the TED clinical trials (7, 8, 10). These abnormalities appear to have multiple etiologies, primarily consequences of either inner ear or eustachian tube dysfunction resulting both in sensorineural and conductive hearing loss (9-11). Accordingly, multiple mechanisms for these abnormalities have been proposed. Since teprotumumab's approval, several observational reports of hearing-related issues have appeared (12-14). The majority of these reports fail to include baseline hearing assessments, and few characterized hearing dysfunction type or severity. These limitations are notable since patients with GD and TED are at increased risk for hearing abnormalities likely related to their underlying thyroid disease regardless of additional treatments or exposures (15-17). Thus, the accurate prevalence of hearing abnormalities directly associated with teprotumumab exposure remains uncertain, with little relevant published information derived from earlier oncology trials with teprotumumab and other IGF-IR inhibitors (18). A prospective study of patients with TED that included standardized audiometry testing before and after teprotumumab therapy found that hearing loss was rare in patients with normal baseline audiometry (19). Further, the incidence of hearing dysfunction among the TED patients at baseline was greater than the general US population matched for age.

In the present study, we conducted analyses of information in a claims database to better understand the hearing adverse event (AE) background rate in GD and TED patients. The prevalence of hearing-related symptoms is determined from patients parsed into those with GD, those with TED, and patients receiving teprotumumab therapy, all of whom have background GD, as mutually exclusive groups for comparison. Further, we analyzed individual patient data from all completed teprotumumab clinical trials, including oncology studies (published and unpublished), for hearing AE incidence, severity, and drug exposure. The findings from these analyses were compared with hearing AEs reported in the teprotumumab trials for TED. QOL outcomes for individuals without and with hearing AEs, as a surrogate for benefit/risk, were examined in these teprotumumab trials of acute TED. Finally, due to the historic classification of TED as either chronic (longer duration, lower activity) or acute (shorter duration, higher activity), we examined if there was a directionality of hearing-related AEs. Patients with chronic TED in a recent, placebo-controlled teprotumumab trial were examined for hearing AE incidence and type.

## Materials and Methods

### Analysis of Ear or Hearing-related Claims

International Classification of Diseases, Tenth Revision (ICD-10) ear and hearing-related codes were examined from deidentified closed claims data (KOMODO Health) (20) covering code entry between September 30, 2015 to August 5, 2022. Three mutually exclusive patient cohorts were assessed (Supplementary Fig. S1) (21): (1) patients with a GD diagnosis without TED. Index date was the first date of GD diagnosis; (2) patients with TED not treated with teprotumumab. Since there is no ICD-10 code specific for TED, a composite of a GD diagnosis and eye manifestations was used, as almost 90% of patients with TED have underlying GD or hyperthyroidism; patients with a GD diagnosis plus eye manifestations of proptosis, diplopia, lid retraction, strabismus (misalignment), and/or orbital inflammation/disorders (ie, “TED patients”) were included. The GD diagnosis and eye manifestation were necessarily within 1 year of each other and the index date was the latter of the two; and (3) patients treated with teprotumumab: patients with 1 or more claims for a teprotumumab infusion as a procedure code (J-code J3241; ie, “teprotumumab patients”). Index date was the date of the first teprotumumab infusion.

Individual patients categorized as “TED patients” were excluded from the “GD patient” cohort. “Teprotumumab patients” were excluded from both “GD patient” and “TED patient” cohorts. Therefore, only patients in the teprotumumab cohort had been treated with teprotumumab. All patients were in the database for 1 year or more prior to and 6 months following entry of their first GD or eye manifestation codes, or 1 year or more prior to and 6 months following their first teprotumumab infusion code. The specific codes used to identify the GD and TED cohorts are provided in Supplementary Table S1 (21). Any ear-related codes were further categorized as 1) hearing loss/ototoxicity codes; 2) other ear and labyrinth codes (including congenital malformations, chromosomal abnormalities, diseases of the inner ear, and injury-related codes); and 3) other ear codes (excluding loss/reduced/altered hearing). These specific ear codes are provided in Supplementary Table S2 (21). Data on ear-related claims, both before and following diagnosis or infusion codes, are included unless indicated otherwise.

### Evaluation of Ear and Labyrinth Disorder Adverse Events in the Teprotumumab Oncology Clinical Trials

The incidence, severity, and timing of ear-related AEs, as classified by the Medical Dictionary for Regulatory Activities (MedDRA), were examined in teprotumumab-treated patients from oncology clinical trials. Details of these trials are provided in Supplementary Table S3 (21). Patients receiving teprotumumab as monotherapy or in combination with other antineoplastic therapy were included. The relation of AEs, judged by each investigator as due to the study drug (teprotumumab or teprotumumab in combination therapy), including severity and drug exposure up to the time of each AE (cumulative mg/kg up to first event), were included.

### Evaluation of Hearing-related Adverse Events in Thyroid Eye Disease Teprotumumab Clinical Trials

Patients treated with teprotumumab from phase 2 (7) (NCT01868997) and phase 3 (8) (OPTIC, NCT03298867) pivotal trials for TED FDA approval and the OPTIC extension (9) (OPTIC-X, NCT03461211) were included in the final analysis. These studies have been described previously (9, 10). Briefly, patients with a TED diagnosis aged 18 to 75 years in the phase 2 trial (7) or 18 to 80 in OPTIC (8) with recent onset (≤9 months’ TED duration) TED with CAS ≥ 4, and OPTIC placebo nonresponders who initially received teprotumumab during OPTIC-X (9) (extension trial) were included here. Placebo and teprotumumab nonresponders from OPTIC could receive open-label treatment by entering OPTIC-X. Patients from phase 2, OPTIC, and OPTIC-X received 8 intravenous infusions of teprotumumab (10 mg/kg body weight for the first infusion, 20 mg/kg for subsequent infusions) every 3 weeks with the treatment phase of the study ending at 24 weeks, 3 weeks after the final infusion. Investigator-reported AEs were collected and coded per MedDRA, version 20.1, and the specific type, onset, and resolution of treatment-emergent, hearing-related AEs were examined. Hearing-related AEs were defined as tinnitus, hearing loss/impairment, hyper/hypoacusis, autophony, and eustachian tube dysfunction. QOL was measured and compared using the Graves’ Ophthalmopathy Quality of Life questionnaire (GO-QOL) (22) scores (range 0-100, with higher scores indicating better QOL) for patients without or with hearing-related AEs during the 24-week trial period.

Data from a phase 4 (23) (NCT04583735) trial of teprotumumab in adults with chronic TED (duration 2-10 years) were also evaluated for hearing AEs. Patients in this trial required a CAS less than or equal to 1, without additional inflammation or progression in proptosis or diplopia for 1 year or longer, and proptosis 3 mm or greater from premorbid observation or from normal limits for race and sex. The dosing regimen and trial design was identical to the phase 2 and OPTIC trials. Investigator-reported treatment-emergent, hearing-related AEs and severity were collected and coded per MedDRA, version 24.0.

Descriptive calculations of incident rates were performed. Baseline differences among the TED trial patients were assessed using *t* tests or chi-square tests as appropriate.

Institutional review board/ethics committee approval and informed consent were obtained for these clinical trials. This research adhered to the tenets of the Declaration of Helsinki. For the KOMODO claims database analysis, only deidentified, aggregate data were used; therefore, institutional review board approval was not required.

## Results

### Ear-related Claims Analyses Among Patients With Graves’ Disease, Patients With Thyroid Eye Disease, and Patients Treated With Teprotumumab

**Figure 1.**
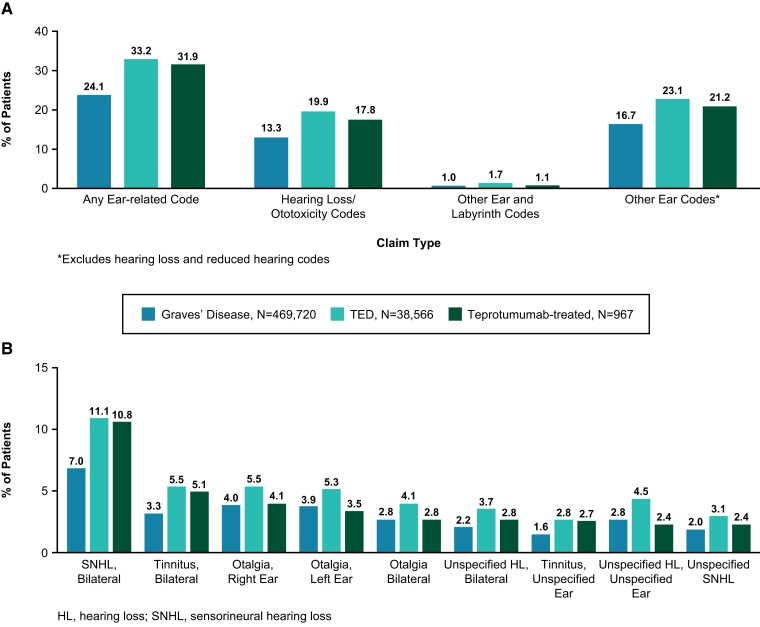
Ear-related claims among Graves’ disease, thyroid eye disease (TED), and teprotumumab patients before and/or after diagnosis or treatment, A, total ear-related claims, and B, most-frequent specific claims.

From the claims data analysis, 469 720 GD patients, 38 566 TED patients, and 967 teprotumumab patients were identified. In each cohort, 73% to 76% were female, with mean age ranging from 52 to 54 years. A similar proportion of GD, TED, and TED patients receiving teprotumumab had any ear-related claims. Any ear-related claims (assessed at any time, both before and/or after index) were reported in 24% of GD patients, 33% of TED patients, and 32% of teprotumumab-treated patients (Fig. 1A). Hearing loss and ototoxicity codes were reported in 13.3% of GD patients, 19.9% of TED patients, and 17.8% of teprotumumab-treated patients. More specifically, sensorineural hearing loss was most frequently encountered among ear-related claims across all 3 cohorts: 7% (32 961/469 720) in the GD cohort, 11.1% (4279/38 566) in the TED cohort, and 10.8% (104/967) in the teprotumumab cohort (Fig. 1B).

**Figure 2.**
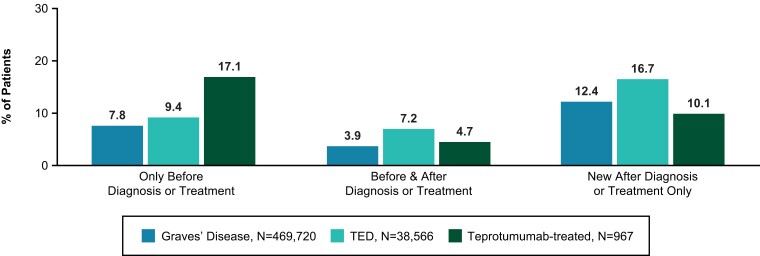
Ear-related claims relative to Graves’ disease diagnosis, thyroid eye disease diagnosis, or first teprotumumab infusion.

Of 469 720 patients with a GD diagnosis, 7.8% (36 562) patients had ear-related claims only prior to GD diagnosis, 3.9% (18 463) had claims both before and after GD diagnosis, and 12.4% (58 243) had claims only following their GD diagnosis (Fig. 2). Of 38 566 patients with a TED diagnosis, 9.4% (3607) had an ear-related claim only prior to that TED diagnosis, 7.2% (2770) had claims both before and after a TED diagnosis, and 16.7% (6430) had claims only following diagnosis. Of 967 patients who received teprotumumab, 17.1% (165) had an ear-related claim only prior to their first infusion, 4.7% (45) had claims both before and after, and 10.1% (98) had claims only after their first teprotumumab infusion.

### Ear and Labyrinth Disorder Adverse Events in Teprotumumab Oncology Clinical Trials

Patients from 8 oncology clinical trials (N = 633) were included, with 362 patients treated with teprotumumab as monotherapy and 271 treated with teprotumumab combined with other antineoplastic agents. These include erlotinib, letrozole, gemcitabine, paclitaxel, bevacizumab, carboplatin, etoposide, FOLFOX6 regimen, capecitabine, trastuzumab, and sorafenib (NCT00400361, NCT00642941, NCT00811993, NCT00760929, NCT00773383, NCT00796107, NCT00985374, NCT00882674; Supplementary Table S3) (21).

**Figure 3.**
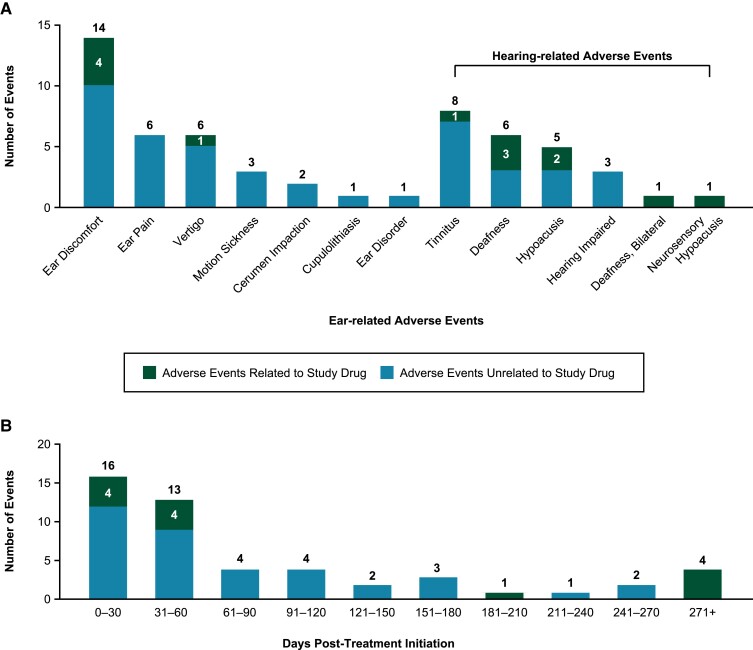
Ear-related adverse events (AEs) among teprotumumab-treated oncology trial patients. A, Ear and labyrinth disorder AEs, N = 57, and B, timing of events reported in teprotumumab-treated oncology trial patients; N = 50 includes events of 13 patients with study drug-related AEs. Of 51 patients with ear and labyrinth disorders, 1 AE of motion sickness missing details. For the 6 patients experiencing 2 ear and labyrinth disorder AEs, the first event is assessed and included in these data.

The ear and labyrinth disorder AE rate was similar among those without and with concomitant therapies; therefore, all patient findings are reported in aggregate. Of the 633 patients from these oncology trials, 8.1% (n = 51) reported 57 ear and labyrinth disorder events (Fig. 3A). Among these 51 patients, 6 experienced 2 different ear and labyrinth disorder events.

Thirteen (2.1%) of these 633 patients reported ear and labyrinth disorder events that were investigator-adjudicated as being study drug related. More specifically, 3.8% (n = 24) reported hearing-related events with 1.3% (n = 8) having hearing-related, study-drug-related AEs. Fifty-eight percent (29/50) of patients reported these ear and labyrinth AEs within 60 days of therapy initiation and 82% (46/56) were considered mild, with ear discomfort being the most frequent (Fig. 3B); this analysis excludes one patient who experienced an AE of motion sickness missing timing, exposure, and severity details.

The frequency and characteristics of ear and labyrinth disorder AEs were similar regardless of their relatedness to the study drug or whether teprotumumab was administered as monotherapy or combined with other antineoplastic agents, some of which have been associated with hearing-related AEs.

**Figure 4.**
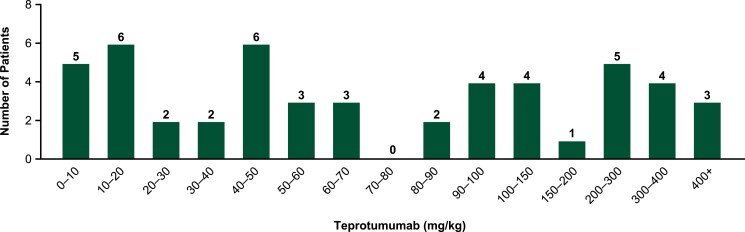
Cumulative teprotumumab exposure of oncology trial patients with ear and labyrinth disorder adverse events (AEs). N = 50, 1 AE of motion sickness missing details to determine exposure. For the 6 patients experiencing 2 ear and labyrinth disorder AEs, the first event is assessed and included in these data.

Most patients reporting ear and labyrinth disorder AEs with teprotumumab from the oncology trials had similar drug exposure to the current FDA-recommended 150 mg/kg cumulative dosing regimen for TED, with most events being mild across all doses (Fig. 4 and Table 1). Of the 13 study-drug-related AEs, 2 were considered moderate (deafness and hypoacusis), 1 was severe (bilateral deafness), and the remainder were rated as mild. The severe event occurred in a patient exposed to a cumulative dose of 1008 mg/kg, nearly 7 times the current FDA-recommended TED dosing exposure.

**Table 1.** Severity across exposures of oncology teprotumumab trial patients with ear and labyrinth disorder adverse events

Exposure	Adverse event severity	Patient count
<150 mg/kg	Mild	31
	Moderate	5
	Severe	1
≥150 mg/kg	Mild	10
	Moderate	2
	Severe	1

N = 50, 1 AE of motion sickness missing details to determine exposure. For the 6 patients experiencing 2 ear and labyrinth disorder AEs, the first event is assessed and included in these data.

Abbreviation: AE, adverse event.

### Hearing Events in Teprotumumab-treated Acute Thyroid Eye Disease Trial Patients

Among patients with TED from the teprotumumab phase 2 and OPTIC trials, 12 out of 121 patients (9.9%) reported 13 treatment-emergent, hearing-related AEs (Table 2). Eleven AEs were reported by investigators as resolved/recovered at time of follow-up. Most of the AEs (77%, 10/13) were considered mild and none was severe (Table 3). Six of 13 hearing-related AEs reported across 5 trial sites were considered drug related by the reporting investigator. All patients with hearing-related AEs completed the 24-week study. No placebo-treated patients reported hearing-related AEs in either the phase 2 or OPTIC placebo-controlled trials.

**Table 2.** Unique patients with treatment-emergent hearing-related adverse events in 3 clinical trials of acute thyroid eye disease

	Phase 2	OPTIC	OPTIC-X (1st-course teprotumumab patients)	Total (phase 2, OPTIC, OPTIC-X; 1st-course teprotumumab patients)
Patients with hearing-related events, n (%)	3 (7)	5 (12)	4 (11)	12 (10)
Patients without hearing-related events, n (%)	40 (93)	36 (88)	33 (89)	109 (90)
Total, N	43	41	37	121

**Table 3.** Treatment-emergent hearing-related adverse events in teprotumumab-treated patients in 3 clinical trials of acute thyroid eye disease

Patient	MedDRA preferred term for AE	Severity/Seriousness	Study day onset	Outcome/Relatedness
OPTIC patient 1 male	Hypoacusis	Moderate, nonserious	Day 108	Recovered: day 283, related
OPTIC patient 2*^a^* female	Deafness	Moderate, nonserious	Day 134	Recovered: day 337, related
OPTIC patient 3*^b^* female	Autophony	Mild, nonserious	Day 84	Recovered: ∼4 mo after last dose, related
OPTIC-X patient 1 female	Tinnitus Tinnitus	Mild, nonserious Mild, nonserious	Day 1 Day 84	Recovered: day 20, not related Not recovered, related
OPTIC-X patient 2 female	Tinnitus	Mild, nonserious	Day 69	Recovered, related
Phase 2 patient 1 male	Deafness	Mild, nonserious	At time of 8th infusion	Not recovered, possibly related
Phase 2 patient 2 female	Hyperacusis	Mild, nonserious	Day 84	Recovered: day 251, not likely related
Phase 2 patient 3 female	Eustachian tube dysfunction	Moderate, nonserious	Day 47	Recovered: Day 68, Unrelated
OPTIC patient 4 female	Hypoacusis	Mild, nonserious	Day 75	Recovered: day 76, not related
OPTIC patient 5 female	Eustachian tube patulous	Mild, nonserious	Day 153	Recovered: day 304, Unrelated
OPTIC-X patient 3 female	Hypoacusis	Mild, nonserious	Day 127	Recovered, unrelated
OPTIC-X patient 4 female	Hypoacusis	Mild, nonserious	Day 66	Recovered, unrelated

Abbreviations: AE, adverse event; MedDRA, Medical Dictionary for Regulatory Activities.

^*a*^Experienced hypoacusis during teprotumumab therapy that was mild and ongoing.

^*b*^Experienced autophony during teprotumumab therapy that was mild and ongoing.

TED study patients treated in the phase 2 and OPTIC trials who had hearing-related AEs were 7.5 (mean) years older and had a shorter duration of GD than those without hearing abnormalities (Table 4); however, only the GD duration was significantly different. Other baseline characteristics were comparable to those patients without hearing-related AEs.

**Table 4.** Baseline characteristics of teprotumumab-treated patients with and without treatment-emergent hearing-related adverse events in 3 clinical trials of acute thyroid eye disease

Teprotumumab-treated trial patients	With hearing-related events (N = 12)	Without hearing-related events (N = 109)	*P*
Age, mean (SD), y	57.3 (13.94)	49.8 (11.87)	.106
Female, n (%)	10 (83.3)	75 (68.8)	.225
Graves’ disease duration, mean (SD), y	1.64 (1.83)	3.17 (4.88)	.016
TED duration, mean (SD), mo	8.28 (3.11)	7.80 (3.79)	.763
Proptosis, mean (SD), mm	22.21 (2.42)	23.09 (3.23)	.232
Diplopia score*^a^*, mean (SD)	1.33 (1.23)	1.56 (1.15)	.344
CAS, mean (SD)	4.58 (1.44)	4.65 (1.37)	.914
GO-QOL overall score, mean (SD)	61.04 (15.95)	62.90 (21.58)	.791
GO-QOL appearance score, mean (SD)	57.81 (24.88)	59.00 (26.28)	.817
GO-QOL visual function score, mean (SD)	64.36 (25.75)	66.69 (25.81)	.810

Abbreviations: CAS, clinical activity score; GO-QOL, Graves’ Ophthalmopathy Quality of Life questionnaire; TED, thyroid eye disease.

^*a*^Gorman Subjective Diplopia Score, range 0 to 3 (24).

**Figure 5.**
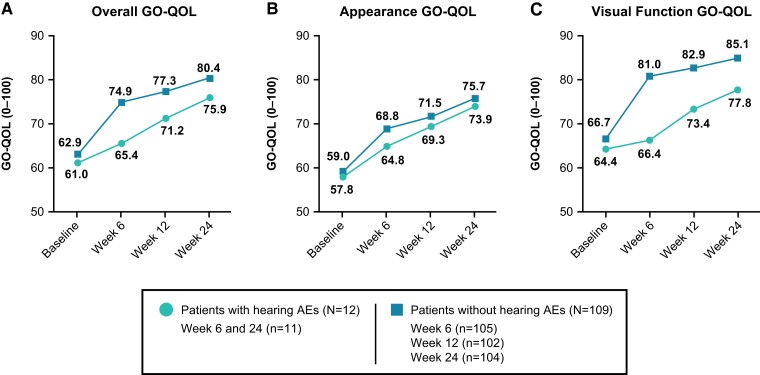
A, Mean overall Graves’ Ophthalmopathy Quality of Life Questionnaire (GO-QOL) scores; B, Mean appearance GO-QOL subscale scores; and C, Mean visual function subscale scores of teprotumumab-treated patients with acute thyroid eye disease with (n = 12) and without (n = 109) hearing-related adverse events (AEs).

Patients with hearing-related AEs demonstrated similar improvements in GO-QOL with teprotumumab therapy to those without them (Fig. 5). At the end of the treatment period, 3 weeks after the final infusion of teprotumumab, the mean overall GO-QOL score was 80.4 and 75.9 for those without and with hearing-related AEs, respectively. These scores indicate moderate to large improvement (22) from mean baseline scores (by 17.5 and 15 points, respectively). Notably, every patient with a hearing AE experienced an improvement from their baseline GO-QOL score. This trend is reflected both in mean appearance and visual function subscales, with improvements of similar magnitude from baseline as patients without hearing AEs.

Patients with chronic TED experienced a slightly higher incidence of hearing-related AEs than those reported in the earlier trials of teprotumumab for acute TED but a similar rate compared to the placebo group in previous trials; 22% for teprotumumab and 10% for placebo patients (Table 5) (23). In the teprotumumab group, 9 patients reported 12 hearing-related AEs. These patients were on average 4.7 years older than the teprotumumab-treated trial patients without hearing-related AEs, although this was not statistically different. One serious AE of conductive deafness occurred in a patient with a congenital anomaly; this patient completed all 8 infusions. In the placebo group, 2 patients reported 3 AEs (tinnitus). Similar to the phase 2 and OPTIC trials, hypoacusis was the most common treatment-emergent, hearing-related AE.

**Table 5.** Hearing-related adverse events among teprotumumab-treated patients with chronic thyroid eye disease (20)

	Teprotumumab (N = 41)		Placebo (N = 20)^a^	
	Patients, n (%)	Events	Patients, n (%)	Events
Hearing impairment	9 (22.0)	12	2 (10.0)	3
Autophony	1 (2.4)	1	—	
Conductive deafness^b^	1 (2.4)	1	—	
Deafness unilateral	1 (2.4)	2	—	
Eustachian tube dysfunction	1 (2.4)	1	—	
Hypoacusis	4 (9.8)	4	—	
Tinnitus	2 (4.9)	2	2 (10.0)	3
Tympanic membrane disorder	1 (2.4)	1	—	

^*a*^Includes one patient who received one infusion of teprotumumab in error.

^*b*^Conductive hearing loss in patient with congenital hearing abnormality.

## Discussion

These data expand our understanding of hearing-related AEs associated with GD and TED. Studies examining patients with GD but not treated with teprotumumab have revealed decreased hearing ability, especially at high frequencies, with mild-moderate sensorineural hearing loss noted in as many as 23.5% of patients (15, 16). Observations ascertained from claims-based data reported here demonstrate comparable or lower rates of hearing-related medical claims among patients receiving teprotumumab when compared with GD patients without or with TED not receiving teprotumumab.

Despite the oncology programs for teprotumumab being terminated for lack of efficacy, no major drug safety concerns were identified. This sizable oncology clinical trial program does, however, provide additional data on hearing-related AEs at an incidence comparable to that found in the TED clinical trial program and was included in the current FDA-approved prescribing label for teprotumumab. Moreover, the hearing AE types and incidence were similar in patients receiving teprotumumab as monotherapy or when combined with other antineoplastic agents. Most hearing AEs were mild. The few moderate and severe AEs judged related to the study drug were mostly at higher teprotumumab exposures than those used in the TED clinical trials or currently recommended for clinical practice. The types of AEs and incidence were consistent with those observed in the TED clinical trials.

In our analysis of the phase 2 and OPTIC clinical trials involving patients with TED, none of the participants reporting a hearing AE withdrew from treatment. All patients completed the study, and all reported clinically meaningful GO-QOL improvement with teprotumumab treatment, similar to those not reporting hearing AEs. QOL in these teprotumumab-treated patients continued to improve over time. Assessment of patients with chronic TED indicates a fractionally higher incidence of mild hearing-related AEs, but a similar difference versus placebo patients, than the earlier trials with acute TED. Other studies have reported that older patients are at increased risk for hearing-related dysfunction, regardless of whether they have been treated with teprotumumab (19, 25).

The increased propensity of hearing-related AEs has been reported in recent case series of teprotumumab-treated patients (19, 25). A prospective study disclosed that those exhibiting hearing-related changes with teprotumumab more commonly had abnormal baseline audiograms, were typically older, and had higher baseline CAS (2.8 vs 2) compared to those not reporting hearing-related changes (19). Another case series of 22 patients, all of whom had undergone both baseline and posttreatment audiograms, revealed that 11 of 22 patients (50.0%) met criteria for ototoxicity in at least 1 ear; however, the severity of these AEs was not reported (25). Pure tone average hearing levels in both ears of every patient revealed that 20 of 44 ears exhibited posttreatment hearing loss that was specific for high (*P* = .0008) and mid-frequencies (*P* = .0042), but that low frequency hearing was unaffected. This study also suggested that older patients were more likely to experience hearing loss (*P* = .0048) (25). Importantly, neither of these analyses found any correlation between objective hearing dysfunction and reported subjective AEs, underscoring the necessity for formal hearing acuity assessments. Moreover, many people have preexisting hearing loss, a risk factor for progression during treatment, without being aware of it (11). A recent publication used the FDA Adverse Event Reporting System (FAERS) database to assess potential safety signals of teprotumumab with review of postmarketing safety data (26). However, it should be emphasized that these reports were not validated by health-care professionals and “duplicate and incomplete reports are in the system” (27). Further, the information in these reports cannot be used to estimate the incidence (occurrence rates) of the events reported.

### Limitations

Due to the inherent limitations of claims data, neither history nor outcomes of these claims could be followed longitudinally. Further, neither causality to the underlying disease nor relationship to drug therapy could be established, including whether the medical claim for “ototoxicity” met standard formal diagnostic criteria. Our present analysis does not capture patients whose hearing could have improved during or after therapy, which has been reported previously (25). Moreover, many patient details, context of severity (as reported in the clinical trial data), and follow-up data are unavailable for claims data. In the oncology teprotumumab trials, AE follow-up is similarly not available. Although the rate of ear and labyrinth disorder and hearing AEs in patients without or with concomitant antineoplastic therapy in the oncology trials did not differ, we cannot determine whether other drugs known to affect hearing, administered in combination with teprotumumab, might not have increased the rate of hearing AEs. Identifying risk factors associated with hearing events after teprotumumab therapy would also be clinically valuable information, but the small number of patients in the TED trials manifesting hearing AEs precluded this assessment, though there are data available from a published prospective trial on this (19). Finally, differences exist between the coding in claims data and the MedDRA categorization used in the clinical trial data; however, reporting both provides a complimentary assessment of hearing-related AE reports.

## Conclusion

Therapy-emergent, hearing-related AEs necessitate objectively quantifying baseline hearing function and frequent follow-up during and after treatment with teprotumumab. It is strongly recommended that any risk factors, including advanced age, ototoxic noise, other drug exposure, and baseline hearing abnormalities be identified and discussed with each patient prior to initiating therapy. Weighing potential benefit and risk of therapy for patients with TED, a lifelong-altering disease, is warranted.

It becomes evident from these analyses that assessment of baseline hearing-related issues is a necessary component of medical care in patients with thyroid autoimmunity prior to ascribing any changes to teprotumumab exposure. Given the evolving nature of reports of hearing AEs assigned to teprotumumab, especially in those with existing hearing impairment, it is prudent for clinicians to assess patients’ hearing before, during, and after treatment with teprotumumab and discuss the benefit-risk of treatment with patients, as currently recommended in the prescribing information for the product (28).

## Data Availability

There is a plan to share data. This may include deidentified individual patient data for variables necessary to address the specific research question in an approved data-sharing request; also related data dictionaries, study protocol, statistical analysis plan, informed consent form, and/or clinical study report. Data-sharing requests relating to data in this manuscript will be considered after the publication date and 1) this product and indication (or other new use) have been granted marketing authorization both in the United States and Europe, or 2) clinical development discontinues and the data will not be submitted to regulatory authorities. There is no end date for eligibility to submit a data-sharing request for these data. Qualified researchers may submit a request containing the research objectives, the Amgen product(s) and Amgen study/studies in scope, end points/outcomes of interest, statistical analysis plan, data requirements, publication plan, and qualifications of the researcher(s). In general, Amgen does not grant external requests for individual patient data for the purpose of reevaluating safety and efficacy issues already addressed in the product labeling. A committee of internal advisors reviews requests. If not approved, requests may be further arbitrated by a data-sharing independent review panel. Requests that pose a potential conflict of interest or an actual or potential competitive risk may be declined at Amgen's sole discretion and without further arbitration. On approval, information necessary to address the research question will be provided under the terms of a data-sharing agreement. This may include anonymized individual patient data and/or available supporting documents, containing fragments of analysis code where provided in analysis specifications. Further details are available at the following: https://wwwext.amgen.com/science/clinical-trials/clinical-data-transparency-practices/clinical-trial-data-sharing-request.

## Prior Presentation

Some of this work was presented at the American Academy of Clinical Endocrinology Annual Meeting 2023 and the Endocrine Society Annual Meeting (ENDO) 2023.

## Prior Affiliation

Amgen Inc was formerly Horizon Therapeutics plc, Deerfield, Illinois, USA.

## Abbreviations

AE: adverse event
CAS: clinical activity score
FDA: US Food and Drug Administration
GD: Graves’ disease
GO-QOL: Graves’ Ophthalmopathy Quality of Life questionnaire
ICD-10: International Classification of Diseases, Tenth Revision
IGF-IR: insulin-like growth factor-1 receptor
MedDRA: Medical Dictionary for Regulatory Activities
QOL: quality of life
TED: thyroid eye disease
